# (*E*)-*N*′-(2-Furylmethyl­ene)benzo­hydrazide

**DOI:** 10.1107/S1600536809042251

**Published:** 2009-10-23

**Authors:** Ming-Zhi Song, Chuan-Gang Fan

**Affiliations:** a College of Chemistry and Chemical Technology, Binzhou University, Binzhou 256600, Shandong, People’s Republic of China

## Abstract

In the title compound, C_12_H_10_N_2_O_2_, the dihedral angle between the benzene and furan rings is 52.54 (7)°. In the crystal, inter­molecular N—H⋯O hydrogen bonds and C—H⋯π inter­actions link the mol­ecules.

## Related literature

For biological properties of Schiff base ligands, see: Chakraborty *et al. *(1996 ▶); Jeewoth *et al. *(1999 ▶). For related crystal structures, see: Fun *et al. *(2008 ▶); Cui *et al. *(2009 ▶); Nie (2008 ▶).
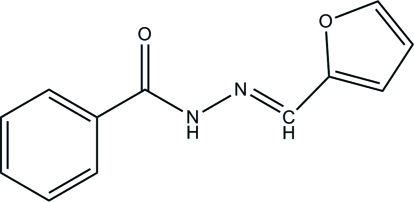


## Experimental

### 

#### Crystal data

C_12_H_10_N_2_O_2_
*M* *_r_* = 214.22
Monoclinic, 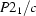

*a* = 12.3955 (11) Å
*b* = 9.4777 (9) Å
*c* = 9.6845 (10) Å
β = 110.610 (1)°
*V* = 1064.93 (18) Å^3^
*Z* = 4
Mo *K*α radiation
μ = 0.09 mm^−1^
*T* = 298 K
0.43 × 0.38 × 0.30 mm

#### Data collection

Bruker SMART APEX CCD area-detector diffractometer
Absorption correction: multi-scan (*SADABS*; Sheldrick, 1996 ▶) *T*_min_ = 0.961, *T*_max_ = 0.973
5190 measured reflections
1882 independent reflections
1360 reflections with *I* > 2σ(*I*)
*R*_int_ = 0.032

#### Refinement

*R*[*F* ^2^ > 2σ(*F* ^2^)] = 0.038
*wR*(*F* ^2^) = 0.103
*S* = 1.05
1882 reflections
145 parameters
H-atom parameters constrained
Δρ_max_ = 0.18 e Å^−3^
Δρ_min_ = −0.20 e Å^−3^

### 

Data collection: *SMART* (Siemens, 1996 ▶); cell refinement: *SAINT* (Siemens, 1996 ▶); data reduction: *SAINT*; program(s) used to solve structure: *SHELXS97* (Sheldrick, 2008 ▶); program(s) used to refine structure: *SHELXL97* (Sheldrick, 2008 ▶); molecular graphics: *SHELXTL* (Sheldrick, 2008 ▶); software used to prepare material for publication: *SHELXTL*.

## Supplementary Material

Crystal structure: contains datablocks I, global. DOI: 10.1107/S1600536809042251/bq2168sup1.cif

Structure factors: contains datablocks I. DOI: 10.1107/S1600536809042251/bq2168Isup2.hkl

Additional supplementary materials:  crystallographic information; 3D view; checkCIF report

## Figures and Tables

**Table 1:** Hydrogen-bond geometry (Å, °)

*D*—H⋯*A*	*D*—H	H⋯*A*	*D*⋯*A*	*D*—H⋯*A*
N1—H1⋯O1^i^	0.86	2.14	2.972 (2)	163
C10—H10⋯*Cg*1^ii^	0.93	2.84	3.498 (2)	128

Symmetry codes: (i) 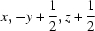; (ii) 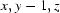. *Cg*1 is the centroid of the C2–C7 ring.

**Fig. 1.**
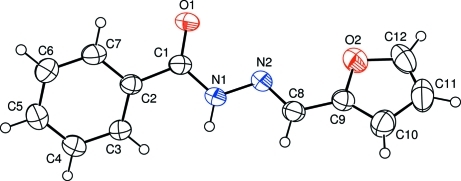
A view of (I) showing the atomic numbering scheme and 50% probability displacement ellipsoids.

**Fig. 2.**
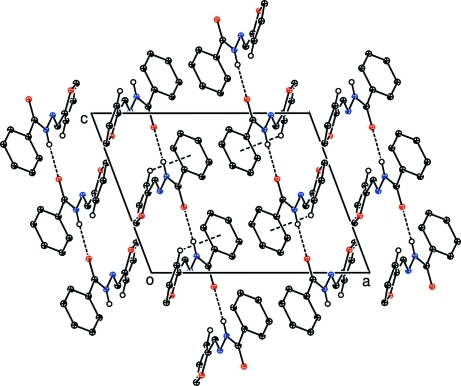
A packing of (I) viewed down b-axis showing the N-H..O and C-H···π H-bond interactions with dashed lines. Symmetry codes: (i) x, -y+1/2, z+1/2; (ii) x, y-1, z.

## supplementary crystallographic information

### Comment

Conventionally, Schiff bases derived from a large number of carbonyl compounds and amines. It has been shown that Schiff base compounds have strong anticancer activity (Chakraborty *et al.*, 1996). It has been well known that a series of certain Schiff base compounds, have received considerable attention during the last decades, mainly because their structures or for their biological properties (Jeewoth *et al.*, 1999).

In the compound (I), (Fig. 1), the bond lengths an angles are normal and are comparable to the values observed in similar compounds (Nie *et al.*, 2008; Fun *et al.*, 2008; Cui *et al.*, 2009).

In the crystal structure, the C=N bond length in the molecule is 1.273 (2) ° (C8=N2), showing the double-bond character. Meanwhile, the dihedral angle between the benzene ring (C2-C7) and the furan ring (C9-C12/O2) in the Schiff base molecule is 52.54 (7)°, indicating that the two aromatic ring planes are not coplanar.

Moreover, the crystal supramolecular structure was built from the connections of intermolecular N—H···O hydrogen bonds and C-H···π hydrogen bonding interactions, as shown in Table 1.

### Experimental

Benzohydrazide (5.0 mmol), 20 ml ethanol and furfural (5.0 mmol) were mixed in 50 ml flash. After refluxing 3 h, the resulting mixture was cooled to room temperature, and recrystalized from ethanol, and afforded the title compound as a crystalline solid. Elemental analysis: calculated for C_12_H_10_N_2_O_2_: C 67.28, H 4.71, N 13.08%; found: C 67.16, H 4.66, N 13.19%.

### Refinement

All H atoms were placed in geometrically idealized positions (N—H 0.86 and C—H 0.93 Å) and treated as riding on their parent atoms, with *U*_iso_(H) = 1.2U5_eq_(C) (C,*N*).

### Crystal data

**Table:**

C_12_H_10_N_2_O_2_	*F*(000) = 448
*M**_r_* = 214.22	*D*_x_ = 1.336 Mg m^−^^3^
Monoclinic, *P*2_1_/*c*	Mo *K*α radiation, λ = 0.71073 Å
Hall symbol: -P 2ybc	Cell parameters from 1760 reflections
*a* = 12.3955 (11) Å	θ = 2.8–25.6°
*b* = 9.4777 (9) Å	µ = 0.09 mm^−^^1^
*c* = 9.6845 (10) Å	*T* = 298 K
β = 110.610 (1)°	Needle, green
*V* = 1064.93 (18) Å^3^	0.43 × 0.38 × 0.30 mm
*Z* = 4	

### Data collection

**Table:**

Bruker SMART APEX CCD area-detector diffractometer	1882 independent reflections
Radiation source: fine-focus sealed tube	1360 reflections with *I* > 2σ(*I*)
graphite	*R*_int_ = 0.032
phi and ω scans	θ_max_ = 25.0°, θ_min_ = 1.8°
Absorption correction: multi-scan (*SADABS*; Sheldrick, 1996)	*h* = −14→14
*T*_min_ = 0.961, *T*_max_ = 0.973	*k* = −11→5
5190 measured reflections	*l* = −11→11

### Refinement

**Table:**

Refinement on *F*^2^	Primary atom site location: structure-invariant direct methods
Least-squares matrix: full	Secondary atom site location: difference Fourier map
*R*[*F*^2^ > 2σ(*F*^2^)] = 0.038	Hydrogen site location: inferred from neighbouring sites
*wR*(*F*^2^) = 0.103	H-atom parameters constrained
*S* = 1.05	*w* = 1/[σ^2^(*F*_o_^2^) + (0.0444*P*)^2^ + 0.2066*P*] where *P* = (*F*_o_^2^ + 2*F*_c_^2^)/3
1882 reflections	(Δ/σ)_max_ < 0.001
145 parameters	Δρ_max_ = 0.18 e Å^−^^3^
0 restraints	Δρ_min_ = −0.20 e Å^−^^3^

### Special details

**Table:**

Geometry. All e.s.d.'s (except the e.s.d. in the dihedral angle between two l.s. planes) are estimated using the full covariance matrix. The cell e.s.d.'s are taken into account individually in the estimation of e.s.d.'s in distances, angles and torsion angles; correlations between e.s.d.'s in cell parameters are only used when they are defined by crystal symmetry. An approximate (isotropic) treatment of cell e.s.d.'s is used for estimating e.s.d.'s involving l.s. planes.
Refinement. Refinement of *F*^2^ against ALL reflections. The weighted *R*-factor *wR* and goodness of fit *S* are based on *F*^2^, conventional *R*-factors *R* are based on *F*, with *F* set to zero for negative *F*^2^. The threshold expression of *F*^2^ > σ(*F*^2^) is used only for calculating *R*-factors(gt) *etc*. and is not relevant to the choice of reflections for refinement. *R*-factors based on *F*^2^ are statistically about twice as large as those based on *F*, and *R*- factors based on ALL data will be even larger.

### Fractional atomic coordinates and isotropic or equivalent isotropic displacement parameters (Å^2^)

**Table:**

	*x*	*y*	*z*	*U*_iso_*/*U*_eq_	
N1	0.23549 (11)	0.22758 (14)	0.10288 (15)	0.0393 (4)	
H1	0.2462	0.2263	0.1956	0.047*	
N2	0.18065 (12)	0.11622 (15)	0.01303 (15)	0.0394 (4)	
O1	0.26227 (11)	0.34211 (13)	−0.08757 (13)	0.0525 (4)	
O2	0.05357 (11)	−0.11197 (14)	−0.14345 (14)	0.0552 (4)	
C1	0.27183 (14)	0.33810 (18)	0.04323 (18)	0.0369 (4)	
C2	0.32908 (13)	0.45449 (17)	0.14650 (18)	0.0357 (4)	
C3	0.31657 (15)	0.47508 (18)	0.28218 (19)	0.0439 (4)	
H3	0.2698	0.4148	0.3122	0.053*	
C4	0.37320 (17)	0.5846 (2)	0.3724 (2)	0.0539 (5)	
H4	0.3642	0.5982	0.4628	0.065*	
C5	0.44318 (17)	0.6740 (2)	0.3290 (2)	0.0576 (6)	
H5	0.4822	0.7468	0.3908	0.069*	
C6	0.45522 (16)	0.6555 (2)	0.1942 (2)	0.0553 (5)	
H6	0.5019	0.7161	0.1646	0.066*	
C7	0.39802 (15)	0.5469 (2)	0.1033 (2)	0.0460 (5)	
H7	0.4057	0.5354	0.0118	0.055*	
C8	0.17622 (14)	0.00187 (18)	0.07975 (19)	0.0407 (4)	
H8	0.2088	−0.0011	0.1821	0.049*	
C9	0.12239 (14)	−0.12242 (18)	0.00150 (19)	0.0406 (4)	
C10	0.12911 (17)	−0.2579 (2)	0.0460 (2)	0.0574 (5)	
H10	0.1701	−0.2924	0.1398	0.069*	
C11	0.06160 (19)	−0.3374 (2)	−0.0779 (3)	0.0693 (6)	
H11	0.0504	−0.4346	−0.0818	0.083*	
C12	0.01745 (18)	−0.2464 (3)	−0.1875 (3)	0.0635 (6)	
H12	−0.0316	−0.2708	−0.2817	0.076*	

### Atomic displacement parameters (Å^2^)

**Table:**

	*U*^11^	*U*^22^	*U*^33^	*U*^12^	*U*^13^	*U*^23^
N1	0.0512 (9)	0.0397 (8)	0.0276 (7)	−0.0026 (7)	0.0147 (6)	−0.0027 (6)
N2	0.0459 (8)	0.0386 (8)	0.0341 (8)	−0.0018 (7)	0.0146 (6)	−0.0038 (7)
O1	0.0821 (9)	0.0482 (8)	0.0309 (7)	−0.0056 (7)	0.0245 (6)	−0.0012 (6)
O2	0.0565 (8)	0.0579 (9)	0.0459 (8)	−0.0045 (7)	0.0116 (6)	−0.0062 (7)
C1	0.0414 (9)	0.0386 (10)	0.0318 (9)	0.0061 (8)	0.0142 (7)	0.0030 (8)
C2	0.0383 (9)	0.0362 (9)	0.0332 (9)	0.0047 (7)	0.0134 (7)	0.0017 (8)
C3	0.0554 (11)	0.0432 (10)	0.0381 (10)	−0.0067 (9)	0.0225 (8)	−0.0012 (8)
C4	0.0699 (13)	0.0579 (13)	0.0391 (11)	−0.0131 (10)	0.0257 (10)	−0.0102 (9)
C5	0.0634 (12)	0.0576 (13)	0.0502 (12)	−0.0191 (11)	0.0180 (10)	−0.0126 (10)
C6	0.0571 (12)	0.0598 (13)	0.0522 (12)	−0.0185 (10)	0.0233 (10)	−0.0014 (10)
C7	0.0509 (11)	0.0534 (11)	0.0386 (10)	−0.0041 (9)	0.0218 (9)	0.0008 (9)
C8	0.0451 (10)	0.0430 (10)	0.0338 (9)	0.0013 (8)	0.0136 (8)	−0.0006 (8)
C9	0.0428 (10)	0.0429 (11)	0.0378 (10)	0.0015 (8)	0.0162 (8)	−0.0012 (8)
C10	0.0647 (13)	0.0460 (12)	0.0627 (13)	0.0011 (10)	0.0241 (11)	0.0039 (11)
C11	0.0781 (15)	0.0457 (12)	0.0926 (19)	−0.0118 (12)	0.0406 (14)	−0.0163 (13)
C12	0.0560 (12)	0.0711 (15)	0.0633 (14)	−0.0171 (12)	0.0210 (11)	−0.0292 (13)

### Geometric parameters (Å, °)

**Table:**

N1—C1	1.348 (2)	C5—C6	1.376 (3)
N1—N2	1.3844 (19)	C5—H5	0.9300
N1—H1	0.8600	C6—C7	1.377 (3)
N2—C8	1.273 (2)	C6—H6	0.9300
O1—C1	1.2306 (19)	C7—H7	0.9300
O2—C9	1.366 (2)	C8—C9	1.432 (2)
O2—C12	1.368 (2)	C8—H8	0.9300
C1—C2	1.490 (2)	C9—C10	1.348 (3)
C2—C7	1.387 (2)	C10—C11	1.415 (3)
C2—C3	1.390 (2)	C10—H10	0.9300
C3—C4	1.379 (2)	C11—C12	1.327 (3)
C3—H3	0.9300	C11—H11	0.9300
C4—C5	1.380 (3)	C12—H12	0.9300
C4—H4	0.9300		
			
C1—N1—N2	119.16 (13)	C5—C6—H6	120.1
C1—N1—H1	120.4	C7—C6—H6	120.1
N2—N1—H1	120.4	C6—C7—C2	120.79 (17)
C8—N2—N1	115.43 (14)	C6—C7—H7	119.6
C9—O2—C12	105.68 (16)	C2—C7—H7	119.6
O1—C1—N1	122.65 (16)	N2—C8—C9	121.81 (16)
O1—C1—C2	121.24 (15)	N2—C8—H8	119.1
N1—C1—C2	116.08 (14)	C9—C8—H8	119.1
C7—C2—C3	118.82 (16)	C10—C9—O2	110.11 (16)
C7—C2—C1	117.59 (15)	C10—C9—C8	130.51 (17)
C3—C2—C1	123.59 (15)	O2—C9—C8	119.36 (15)
C4—C3—C2	120.22 (17)	C9—C10—C11	106.54 (19)
C4—C3—H3	119.9	C9—C10—H10	126.7
C2—C3—H3	119.9	C11—C10—H10	126.7
C3—C4—C5	120.23 (18)	C12—C11—C10	106.65 (19)
C3—C4—H4	119.9	C12—C11—H11	126.7
C5—C4—H4	119.9	C10—C11—H11	126.7
C6—C5—C4	120.03 (18)	C11—C12—O2	111.01 (18)
C6—C5—H5	120.0	C11—C12—H12	124.5
C4—C5—H5	120.0	O2—C12—H12	124.5
C5—C6—C7	119.89 (18)		

### Hydrogen-bond geometry (Å, °)

**Table:**

*D*—H···*A*	*D*—H	H···*A*	*D*···*A*	*D*—H···*A*
N1—H1···O1^i^	0.86	2.14	2.972 (2)	163
C10—H10···Cg1^ii^	0.93	2.85	3.498 (2)	128

Symmetry codes: (i) *x*, −*y*+1/2, *z*+1/2; (ii) *x*, *y*−1, *z*.

## References

[bb1] Chakraborty, J. & Patel, R. N. (1996). *J. Indian Chem. Soc.* **73**, 191–195.

[bb2] Cui, C., Meng, Q. & Wang, Y. (2009). *Acta Cryst.* E**65**, o2472. 10.1107/S1600536809036460 PMC2970421 21577924

[bb3] Fun, H.-K., Patil, P. S., Jebas, S. R., Sujith, K. V. & Kalluraya, B. (2008). *Acta Cryst.* E**64**, o1594–o1595. 10.1107/S1600536808022861 PMC2962208 21203289

[bb4] Jeewoth, T., Bhowon, M. G. & Wah, H. L. K. (1999). *Transition Met. Chem.* **24**, 445–448.

[bb5] Nie, Y. (2008). *Acta Cryst.* E**64**, o471. 10.1107/S160053680800130X PMC2960467 21201497

[bb6] Sheldrick, G. M. (1996). *SADABS* University of Göttingen, Germany.

[bb7] Sheldrick, G. M. (2008). *Acta Cryst.* A**64**, 112–122. 10.1107/S0108767307043930 18156677

[bb8] Siemens (1996). *SMART* and *SAINT* Siemens Analytical X-ray Instruments Inc., Madison, Wisconsin, USA.

